# Two-year outcomes following a randomised platelet transfusion trial in preterm infants

**DOI:** 10.1136/archdischild-2022-324915

**Published:** 2023-02-21

**Authors:** Carmel Maria Moore, Angela D’Amore, Suzanne Fustolo-Gunnink, Cara Hudson, Alice Newton, Beatriz Lopez Santamaria, Alison Deary, Renate Hodge, Valerie Hopkins, Ana Mora, Charlotte Llewelyn, Vidheya Venkatesh, Rizwan Khan, Karen Willoughby, Wes Onland, Karin Fijnvandraat, Helen V New, Paul Clarke, Enrico Lopriore, Timothy Watts, Simon Stanworth, Anna Curley, for the PlaNeT2 MATISSE, Timothy Watts

**Affiliations:** 1 School of Medicine, University College Dublin, Dublin, Ireland; 2 Neonatology, National Maternity Hospital, Dublin, Ireland; 3 Neonatology, Cambridge University Hospitals NHS Foundation Trust, Cambridge, UK; 4 Clinical Transfusion Research, Sanquin, Amsterdam, The Netherlands; 5 Clinical Trials Unit, NHS Blood and Transplant, Bristol, UK; 6 Neonatology, Guy's and St Thomas' NHS Foundation Trust, London, UK; 7 NICU, Cloudnine Hospital, Bengaluru, Karnataka, India; 8 NICU, University Maternity Hospital Limerick, Limerick, Ireland; 9 Neonatology, Amsterdam UMC Locatie AMC, Amsterdam, The Netherlands; 10 Pediatrics, Emma Children’s Hospital, Pediatric Hematology, University of Amsterdam, Amsterdam, The Netherlands; 11 Paediatric Transfusion Medicine, NHS Blood and Transplant, London, UK; 12 Neonatal Intensive Care Unit, Norfolk and Norwich University Hospitals NHS Foundation Trust, Norwich, UK; 13 Norwich Medical School, University of East Anglia, Norwich, UK; 14 Neonatology, Leiden Universitair Medisch Centrum, Leiden, The Netherlands; 15 Neonatal Intensive Care Unit, Guy's and St Thomas' NHS Foundation Trust, London, UK; 16 National Health Service Blood and Transplant, Oxford University Hospitals NHS Foundation Trust, Oxford, UK

**Keywords:** Neonatology, Child Development, Intensive Care Units, Neonatal

## Abstract

**Objective:**

Assess mortality and neurodevelopmental outcomes at 2 years of corrected age in children who participated in the PlaNeT-2/MATISSE (Platelets for Neonatal Transfusion - 2/Management of Thrombocytopenia in Special Subgroup) study, which reported that a higher platelet transfusion threshold was associated with significantly increased mortality or major bleeding compared to a lower one.

**Design:**

Randomised clinical trial, enrolling from June 2011 to August 2017. Follow-up was complete by January 2020. Caregivers were not blinded; however, outcome assessors were blinded to treatment group.

**Setting:**

43 level II/III/IV neonatal intensive care units (NICUs) across UK, Netherlands and Ireland.

**Patients:**

660 infants born at less than 34 weeks’ gestation with platelet counts less than 50×10^9^/L.

**Interventions:**

Infants were randomised to undergo a platelet transfusion at platelet count thresholds of 50×10^9^/L (higher threshold group) or 25×10^9^/L (lower threshold group).

**Main outcomes measures:**

Our prespecified long-term follow-up outcome was a composite of death or neurodevelopmental impairment (developmental delay, cerebral palsy, seizure disorder, profound hearing or vision loss) at 2 years of corrected age.

**Results:**

Follow-up data were available for 601 of 653 (92%) eligible participants. Of the 296 infants assigned to the higher threshold group, 147 (50%) died or survived with neurodevelopmental impairment, as compared with 120 (39%) of 305 infants assigned to the lower threshold group (OR 1.54, 95% CI 1.09 to 2.17, p=0.017).

**Conclusions:**

Infants randomised to a higher platelet transfusion threshold of 50×10^9^/L compared with 25×10^9^/L had a higher rate of death or significant neurodevelopmental impairment at a corrected age of 2 years. This further supports evidence of harm caused by high prophylactic platelet transfusion thresholds in preterm infants.

**Trial registration number:**

ISRCTN87736839.

**WHAT IS ALREADY KNOWN ON THIS TOPIC**

- A higher prophylactic platelet transfusion threshold (50×10^9^/L) in the preterm infant is associated with significantly increased mortality or major bleeding compared with a lower one (25×10^9^/L).

**WHAT THIS STUDY ADDS**

- Preterm infants randomised to a higher platelet transfusion threshold of 50×10^9^/L compared with 25×10^9^/L had a higher rate of death or significant neurodevelopmental impairment at a corrected age of 2 years.

**HOW THIS STUDY MIGHT AFFECT RESEARCH, PRACTICE OR POLICY**

- This further supports evidence of harm caused by high prophylactic platelet transfusion thresholds in preterm infants.

## Introduction

Fifteen million infants are born prematurely worldwide annually.^1^ Despite significant progress in neonatal care over the past decades and improved survival rates, extreme prematurity continues to be associated with long-term impairment, with a high risk of death and major brain injury at early gestations. More than 6% of very low birth weight infants (VLBWIs, birthweight <1500 g) develop cerebral palsy (CP), and 25%–44% have cognitive or behavioural deficits.^2–4^ Major bleeding, especially intraventricular haemorrhage (IVH), is associated with lifetime impairment. Clinicians’ concerns about increased risk of potential bleeding result in many infants with severe thrombocytopenia (platelets <50×10^9^/L) receiving platelet transfusions, despite no evidence that this prevents bleeding and increasing concerns about potential harm.^5 6^

Until recently, the only evidence neonatologists had to support or refute prophylactic platelet transfusion in preterm infants was a single randomised controlled trial (RCT) of higher platelet transfusion thresholds in 1993^7^ and a series of retrospective cohort analyses.^6 8^ From 2011, we conducted a large, international RCT of platelet transfusion thresholds to study short-term and long-term effects of higher, more liberal (50×10^9^/L) versus lower, more restrictive (25×10^9^/L) platelet transfusion thresholds. We found that those randomly assigned to undergo platelet transfusions at a platelet count threshold of 50×10^9^/L had a significantly higher rate of death or major bleeding within 28 days after randomisation than those who received platelet transfusions at a platelet count threshold of 25×10^9^/L.^9^ We also demonstrated that this risk was present irrespective of baseline risk of bleeding or death.^10^

Information on solely short-term outcomes is inadequate to assess the overall benefits and risks of neonatal interventions such as platelet transfusion, potentially affecting lifelong development. We now report results of prespecified longer-term follow-up outcomes in this trial. We aimed to determine whether a higher versus lower threshold for prophylactic platelet transfusion changed the composite rate of death or survival with significant neurodevelopmental impairment at the corrected age of 2 years.

## Methods

The inclusion/exclusion criteria, randomisation procedures and short-term outcomes of the PlaNeT-2/MATISSE randomised trial investigating a liberal versus a restrictive platelet transfusion threshold have been reported previously.^9^ Infants born at <34 weeks’ gestation and without recent major bleeding in the previous 72 hours were eligible for the study if their platelet count dropped below 50×10^9^/L. Between 2011 and 2017, 660 infants were enrolled at 43 centres in the UK, Ireland and The Netherlands and were randomly assigned to undergo platelet transfusions at a higher or lower platelet transfusion threshold. Randomisation was performed using gestational age (GA) and presence of intrauterine growth restriction (IUGR) as minimisation factors.

### Neurodevelopmental outcome

Neurodevelopmental outcomes were assessed at all centres, with Bayley Scale of Infant Development III (BSID-III)^11^ and Griffiths Mental Development Scales–Extended Revised (GMDS-ER).^12^ We considered a score more than 2 SDs below the mean an unfavourable outcome. When these were not available, we contacted parents/guardians directly by phone or post to ask them to complete a parent reporting (Parent Report of Children’s Abilities–Revised (PARCA-R)) assessment.^13^ The PARCA-R^13^ is a parent-completed questionnaire used to assess children’s cognitive and language development at 24 months of age, corrected for gestation, translated into Dutch for infants in The Netherlands. Where formal assessment (BSID-III, GMDS-ER or PARCA-R) could not be obtained, all available information was used to deduce the outcome, including Schedule of Growing Skills II (SGS-II) reports. SGS-II is a screening tool that assesses child development, commonly used in the UK.^14^ Letters from hospital visits and information from hospital consultants, general practitioners, public health nurses, and early intervention services were also reviewed.

For the purposes of analysis, we dichotomised participants as having a favourable or unfavourable outcome, as used in other follow-up studies.^15^ Death was included as a composite with unfavourable neurodevelopmental outcomes to avoid bias due to different redirection of care practices in different neonatal units.^16^ A favourable outcome was given if we could ascertain that a child was alive at 2 years of age corrected for gestation and did not have any of the following: CP that impaired independent walking; global developmental delay assessed by healthcare professionals as >9 months behind expected for age, a pragmatic clinical equivalent of >2 SD below the mean; severe seizure disorder; hearing impairment not corrected by hearing aids; or bilateral visual impairment with no useful vision (light perception only). The assessing paediatricians (AC, AD’A and CMM) used all available information to report outcomes using the 2-year outcome form, a standardised classification of outcomes used in other UK studies.^17^ This pragmatic outcome was prespecified in the original trial protocol, aware of potential difficulties with obtaining formal follow-up on such a large group of children across a high number of sites, especially given moving across networks. When formal assessment scores were available, they were checked for concordance with other available data; however, letters from other healthcare professionals were not requested.

The favourable/unfavourable outcome was determined independently by three clinicians (AC, AD’A and CMM) who reviewed the data submitted by the local centres and were blinded to the treatment arm. In case of disagreement, the decision was made based on two of three assessors’ conclusions. Participants who could not be classified were excluded from the analysis, and reasons for non-classification were tabulated.

### Statistical analysis

All prespecified and preplanned analyses were performed according to the intention-to-treat principle and were included all randomised participants with an available outcome (including those randomised in error). Data were analysed according to the trial arm to which the participant was randomised. The outcome for this follow-up study of death or adverse neurodevelopmental outcome was analysed using a mixed logistic regression model. All models were risk-adjusted for GA and IUGR as covariates (factors used for minimisation) and centre of recruitment as a random effect. All tests were two-sided; a p value of <0.05 was considered statistically significant. SAS software V.9.4 was used to conduct the analyses. This study was powered for the primary outcome of the original study—mortality or major bleed at 28 days post randomisation—not for death or adverse neurodevelopmental outcome at 2 years corrected for gestation.

### Secondary outcomes

The component parts of the composite long-term neurodevelopmental outcome were analysed separately using a mixed logistic regression model.

### Death or dependence on respiratory support at 2 years corrected

As we had found a significant difference in the proportion of participants with bronchopulmonary dysplasia (BPD, defined as respiratory support or oxygen dependence at 36 weeks’ postmenstrual age) between the arms in the main trial, respiratory status at 2 years was of particular interest. Therefore, post hoc exploratory analysis of the proportion of participants who had died or were dependent on oxygen or respiratory support at 2 years of age, corrected for GA, was analysed using a mixed logistic regression model, adjusted for GA and presence of IUGR as covariates, and the centre of recruitment as a random effect.

## Results

### Follow-up and baseline characteristics

A total of 660 infants were enrolled between 2011 and 2017 at 43 centres in the UK, Ireland and The Netherlands. Of the 660 infants recruited to the PlaNet-2 trial, seven were not included in primary outcome analysis. This was due to lack of consent (n=3), rerandomisation errors (n=1) and missing data (no cranial ultrasound) in three infants receiving palliative care at the time of primary outcome assessment. These three infants subsequently died and were included in the 2-year follow-up. Three other infants were excluded as we did not have consent for 2-year follow-up, and 47 infants were completely lost to follow-up. Five children could not be included in our analysis as we had insufficient information to assign a neurodevelopmental outcome.

Baselines characteristics of these 601 children were similar in the two randomised groups at birth (birth weight, GA, antenatal corticosteroids, presence of chorioamnionitis) and at the time of randomisation (incidence of major bleeding including IVH prior to study entry and incidence of sepsis or necrotising enterocolitis (NEC)).^9^

### Primary outcome: death or neurodevelopmental outcome at 2 years of age

**Figure 1:**
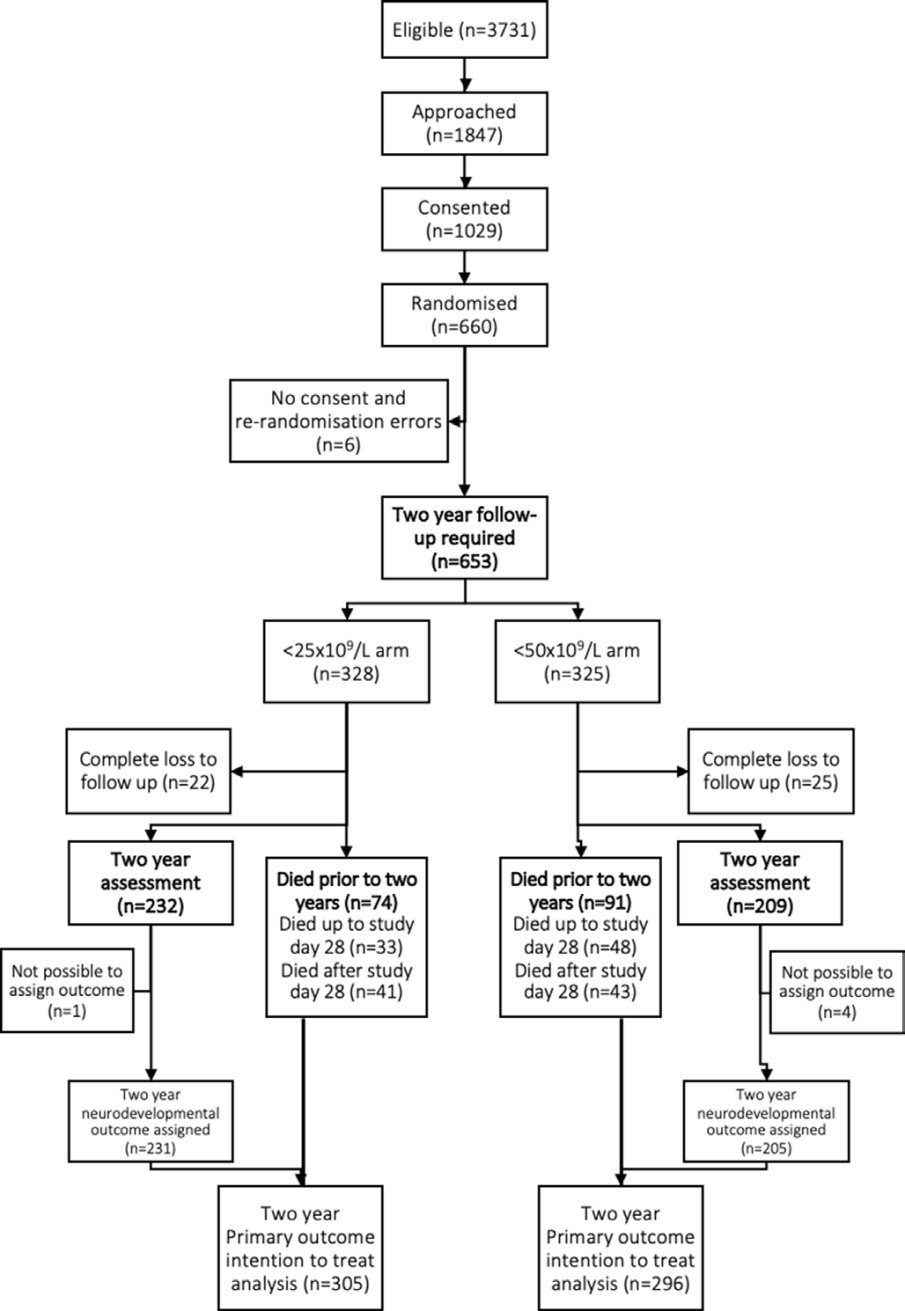
Consolidated Standards of Reporting Trials flow diagram.

Of 653 children, 601 (92%) were available for analysis of the primary composite outcome of death or neurodevelopmental impairment (figure 1). Mortality data were available for 606 of the 653 (93%) infants: 81 infants died within 28 days of recruitment,^9^ and an additional 84 children died before 2 years of corrected age. Five of the surviving children had insufficient data to assign a neurodevelopmental outcome, leaving 436 children available for full neurodevelopmental analysis.

Of 436 children, 177 (41%) had a formal neurodevelopmental assessment, either BSID (158), GMDS (21) or both performed. Of 158 BSID assessments, 134 were sufficient to assign outcome; 15 were incomplete; and 8 were carried out outside of the protocol age range. Of 21 GMDS-ER assessments, 18 met the criteria for assigning an outcome. Of 26 PARCA-R assessments, 3 were sufficient to assign outcome; 4 were incomplete; and 19 were out of the age range. Forty-two children had SGS-II assessment performed. These results are available in online supplemental appendix 1.

10.1136/fetalneonatal-2022-324915.supp1 Supplementary data

Infants randomised to a higher platelet transfusion threshold of 50×10^9^/L compared with 25×10^9^/L had a higher rate of death or significant neurodevelopmental impairment. Of 296 (50%) assigned to the higher threshold group, 147 died or survived with an unfavourable outcome, as compared with 120 of 305 (39%) assigned to the lower threshold group (OR 1.54, adjusted for GA and the presence of IUGR as covariates and centre as a random effect; 95% CI 1.09 to.17; p=0.017), see table 1. The number of infants who would need to be assigned to a lower transfusion threshold to prevent one unfavourable neurodevelopmental outcome was nine (95% CI 5 to 48), based on the risk-adjusted OR and corresponding CI.

**Table 1:** Outcomes

	Low threshold group (n=328)	High threshold group (n=325)	P value
Primary outcome			
Death up to 2 years or unfavourable outcome	120/305 (39)	147/296 (50)	
OR (<50 vs <25) (95% CI)	1.54 (1.09 to 2.17)*		0.0167
Secondary outcomes			
Deaths up to 2 years, n/total n(%)†	74/306 (24)	91/300 (30)	
OR (<50 vs <25) (95% CI)	1.36 (0.93 to 1.99)		0.1067
Unfavourable outcome (excluding deaths), n/total n (%)	46/231 (20)	56/205 (27)	
OR (<50 vs <25) (95% CI)	1.53 (0.95 to 2.46)		0.0788
Participants who have cerebral palsy that impaired independent walking, n/total n (%)‡	23/229 (10)	26/203 (13)	
Participants who have global development delay, n/total n (%)‡	33/228 (14)	44/204 (22)	
Participants who have a severe seizure disorder requiring treatment, n/total n (%)‡	4/225 (2)	7/200 (4)	
Participants who have hearing impairment not corrected by hearing aids, n/total n (%)‡	3/227 (1)	5/203 (2)	
Participants who have bilateral cortical visual impairment with no useful vision, n/total n (%)‡	2/225 (1)	3/200 (2)	
Participants who died or required respiratory support at 2 years of corrected age	83/301 (28)	113/296 (38)	
OR (<50 vs <25) (95% CI)	1.62 (1.12 to 2.34)		
Participants who were dependent on oxygen or respiratory support at 2 years of corrected age	9/228 (4)	22/207(11)	
OR (<50 vs <25) (95% CI)	2.86 (1.25 to 6.51)		

All ORs have been adjusted for GA and presence of IUGR as covariates and centre has been adjusted for using a random effect. Marginal effects are reported.

*The OR has been adjusted for GA and presence of IUGR as covariates and centre has been adjusted for using a random effect. Outcome data were missing for 52 participants (23 in the <25 arm and 29 in the <50 arm). The OR is presented as <50 arm vs <25 arm. Marginal effects are reported.

†Includes five participants who were known to be alive at 2 years, but it was not possible to establish their neurodevelopmental outcome due to insufficient information.

‡Note differing denominators for component parts of neurodevelopmental outcome due to missing data, but unfavourable outcome could still be assigned based on the presence of another adverse factor.

GA, gestational age; IUGR, intrauterine growth restriction.

The higher rates of neurodevelopmental impairment at 2 years consist of a combination of impairments, with global developmental delay and CP being the most common. Fourteen per cent of the lower transfusion threshold and 22% of the higher transfusion threshold group had developmental delay (table 1). CP rates were 10% in the lower threshold group compared with 13% in the higher group. The individual rates of seizure disorder, deafness and blindness at follow-up were similar.

### Secondary outcomes: respiratory

Four of 300 children in the higher threshold group and 5 of 306 children in the lower threshold group had missing data on oxygen dependence at 2 years. Of 296 children assigned to the higher threshold group, 113 (38%) died or required respiratory support at 2 years of age, as compared with 83 (28%) of 301 children assigned to the lower threshold group (OR=1.62, 95% CI 1.12 to 2.34). Twenty-two children in the higher threshold group required respiratory support at 2 years of corrected age compared with 9 children in the lower threshold group. We carried out an additional post hoc analysis to calculate the OR for the participants who were dependent on oxygen or respiratory support at 2 years of corrected age, excluding infants who had died. A statistically significant difference between the trial arms was found in favour of the lower treatment threshold group (OR=2.86, 95% CI 1.25 to 6.51).

## Discussion

We reported previously that a higher threshold for transfusion of 50×10^9^/L increased the rate of mortality or major bleeding 28 days after randomisation in infants born less than 34 weeks’ gestation.^9^ Our finding of potential harm from transfusions is consistent with the results of many observational studies.^5 6 18^ We now report that infants randomised to a higher platelet transfusion threshold of 50×10^9^/L compared with 25×10^9^/L also had a higher composite rate of death or adverse neurodevelopmental outcome at a corrected age of 2 years. Although neither rates of death nor adverse neurological outcome alone differed significantly between trial arms, they both favoured the lower platelet threshold arm. Despite increased survival in the lower threshold cohort, we did not see increased impairment in survivors.

The mechanisms by which platelet transfusions could mediate harmful short-term and long-term effects in the ex-preterm infant remain unknown. It is biologically plausible that platelets could be causing harm through effects on haemodynamics, immunity, inflammation, haemostasis or angiogenesis.^19–22^ These could explain the increase in mortality or affect neurodevelopment. In addition, these mechanisms could potentially cause or aggravate several pre-existing conditions unique to preterm infants including retinopathy of prematurity (ROP), IVH, NEC and BPD, all of which can impact long-term outcomes.^9 18 23–27^ In our study, we identified an increase in IVH and BPD, but not NEC and ROP.^9^

Higher rates of adverse neurodevelopmental and mortality outcomes in the higher-threshold group may be explained by the trend towards an increased rate of severe bleeding up to 28 days and BPD up to 2 years of age. Severe IVH occurred in 11 infants in the higher versus 5 in the lower threshold study arm.^9^ Severe IVH is associated with a high probability of death or impairment, predicting CP and abnormal neurodevelopmental outcome in almost half of infants affected.^28^ BPD is an important cause of mortality beyond the first few months of life^29^ and a very significant predictor of future neurodevelopmental impairment in infancy, childhood and adolescence.^30 31^ There was a trend towards increased rate of death or BPD in the higher threshold group in our study, persisting at 2 years of corrected age.^9^ The mechanisms through which platelet transfusion strategies could cause BPD are unknown but could include inflammation, platelet-neutrophil interactions or neovascularisation due to platelet-derived growth factors.^32–34^ Although BPD occurs in almost one-third of VLBWIs, dependence on respiratory support or oxygen at 2 years of corrected age is uncommon, indicating severe ongoing lung disease.^29^

Infants with NEC and sepsis are frequently thrombocytopenic and are among the highest users of platelet transfusions in the neonatal ICU.^35^ Many of the neonates in our original cohort presented with these conditions causing the thrombocytopenia that led to their inclusion in the study. Platelet transfusion may have proinflammatory effects,^19^ potentially aggravating pre-existing inflammation or need for surgery, thus potentially worsening long-term outcomes in the high threshold group.^9 36–40^

### Strengths and limitations

Few neonatal studies demonstrate significant positive effects on longer-term outcome.^41^ This is the first study assessing long-term effects of platelet transfusion thresholds on neurodevelopmental outcomes in children who were born preterm. We achieved 92% follow-up in our cohort, comparable to other neonatal studies.^42 43^ We chose a pragmatic approach, with crude outcome definition, so that it could be assigned by local paediatricians using a standardised assessment form without formal assessment (if unavailable).^17^ We used standard definitions used in other follow-up studies.^44^ The advantage is that it enabled us to reach high follow-up rates and to assess severe and clinically meaningful outcome data. However, as only 41% of children had formal neurodevelopmental assessment, it was not possible to detect subtle outcome differences. Another limitation is that our study was powered to assess the primary outcome of mortality or major bleed at 28 days post randomisation and not neurodevelopmental or respiratory outcomes at 2 years corrected.

In conclusion, our study found that a higher platelet count threshold of 50×10^9^/L for prophylactic transfusion in preterm infants less than 34 weeks’ gestation at birth increased the rate of death or neurodevelopmental impairment at 2 years of corrected age. There is no evidence to support benefit for high prophylactic platelet transfusion thresholds in the preterm infant. There is increasing evidence of harm persisting into childhood. As clinicians, we need to question whether a liberal approach to platelet transfusion can be justified on clinical or ethical grounds.

## Data Availability

Data are available upon reasonable request. Data will be available upon reasonable request, with approval of the trial group.
