# Supplementary material for: Two-year outcomes following a randomised platelet transfusion trial in preterm infants
Source: Arch Dis Child Fetal Neonatal Ed. 2023 Feb 21;108(5):452–7. doi: 10.1136/archdischild-2022-324915 (PMC10447411; doi:10.1136/archdischild-2022-324915)
Supplement: Supplementary data [file fetalneonatal-2022-324915supp001.pdf]

Appendix Scores from formal neurodevelopmental assessments

Outcome	Low Threshold Group (n=328)		High Threshold Group (n=325)	
	N where formal assessment completed (% of those where 2yr assessment required)	Median (IQR) or n (%)	N where formal assessment completed (% of those where 2yr assessment required)	Median (IQR) or n (%)
**Bayley assessment** [1]	71 (28)		63 (27)	
Cognitive scaled score	67 (26)	10 (5-12)	58 (25)	10 (8-11)
Receptive Language scaled score	48 (19)	9 (7-11)	40 (17)	7 (6-9)
Expressive Language scaled score	50 (20)	8 (5-10)	40 (17)	8 (6-10)
Fine Motor scaled score	63 (25)	10 (5-12)	56 (24)	10 (8-12)
Gross Motor scaled score	61 (24)	8 (6-9)	56 (24)	8 (6-9)
**Griffiths assessment**	8 (3)		10 (4)	
**Locomotor score**	7 (3)		9 (4)	
> 3rd percentile		6 (86)		5 (56)
≤ 3rd percentile		1 (14)		4 (44)
**Personal and Social score**	8 (3)		8 (3)	
> 3rd percentile		6 (75)		6 (75)
≤ 3rd percentile		2 (25)		2 (25)
**Hearing and Language score**	7 (3)		8 (3)	
> 3rd percentile		5 (71)		6 (75)
≤ 3rd percentile		2 (29)		2 (25)
**Eye and Hand Coordination score**	7 (3)		9 (4)	
> 3rd percentile		6 (86)		6 (67)
≤ 3rd percentile		1 (14)		3 (33)
**Performance score**	7 (3)		9 (4)	
> 3rd percentile		5 (71)		5 (56)
≤ 3rd percentile		2 (29)		4 (44)

(1) 8 participants completed a Bayley assessment, but their corrected age was less than 18 months, greater than 36 months, or missing, and so their Bayley assessment was not included in the analysis.
